# Clinical phenotype and karyotype finding of Turner syndrome in Jakarta

**DOI:** 10.1186/1687-9856-2013-S1-P57

**Published:** 2013-10-03

**Authors:** I Nyoman Arie Purwana, I Made Arimbawa, Frida Soesanti, Aman B Pulungan, Jose RL Batubara

**Affiliations:** 1 Department of Pediatrics Endocrinology Cipto Mangunkusumo Hospital, Jakarta, Indonesia; 2 Department of Pediatrics Endocrinology Sanglah Hospital, Denpasar, Indonesia

## 

Turner syndrome (TS) is the most common sex chromosome abnormality of female, occurs in one in 2500 live-born females. TS combines’ characteristic physical features with complete or partial absence of the X chromosomes, frequently accompanied by cell mosaicsm. The aims of this study is to describe the clinical phenotype and karyotype of patient with Turner Syndrome in Jakarta, Indonesia.

Data was collected from medical records of the Pediatrics Endocrinology Clinic, Cipto Mangunkusumo Hospital in Jakarta since 2000-2012.

Of the 23 cases collected, the mean age at diagnosis was 7.75 years (range 0-15 years). The most common problem that bring patients came to the clinic were short stature (69.6%) and delayed puberty (30.4%). There were 8 patients with concomitant disorders: 4 cardiac abnormalities, 3 with ear disorders, 1 with hypertension. From16 patients who had bone age evaluation, 11 patients showed retarded age. A total of 17 patients had a karyotype 45, X and the rest are mosaics.

Our study suggest that the main characteristics of Turner syndrome is a karyotype 45,X with the physical characteristics of short stature and delayed puberty.

